# Expression of Syk and MAP4 proteins in ovarian cancer

**DOI:** 10.1007/s00432-019-02856-9

**Published:** 2019-02-08

**Authors:** Siwei Zhang, Suha Deen, Sarah J. Storr, Anqi Yao, Stewart G. Martin

**Affiliations:** 1 Academic Clinical Oncology, Nottingham Breast Cancer Research Centre, School of Medicine, University of Nottingham, Nottingham University Hospitals NHS Trust, City Hospital Campus, Nottingham, NG5 1PB UK; 2 Department of Pathology, Queen’s Medical Centre, Nottingham University Hospital, Nottingham, NG7 2UH UK

**Keywords:** MAP4, Syk, Calpain, Calpastatin, Ovarian cancer, Chemotherapy

## Abstract

**Purpose:**

We have previously reported on the prognostic importance of the calpain family of proteins in ovarian cancer, especially calpain-2. Spleen tyrosine kinase (Syk) phosphorylates a variety of cytoskeletal proteins with studies suggesting potential interactions between Syk and conventional calpains. Microtubule-associated protein 4 (MAP4) has been reported to be regulated by Syk.

**Methods:**

The current study assessed Syk and MAP4 protein expression, by immunohistochemistry on a tissue microarray comprised of cores from primary ovarian carcinomas (*n* = 575), to evaluate associations with patient clinical outcomes and other clinicopathological factors and sought to determine whether there were any correlations between the expression of Syk, MAP4 and the calpain system.

**Results:**

MAP4 expression was significantly associated with ovarian cancer histological subtype (*P* < 0.001), stage (*P* = 0.001), grade (*P* < 0.001) and residual tumour (*P* = 0.005). Despite this finding, we found no significant association existing between MAP4 expression and overall survival. Syk expression was also found significantly associated with histological subtype (*P* < 0.001). Syk seems to play a contradictory role with respect to tumour progression: low cytoplasmic Syk expression was significantly associated with low stage (*P* = 0.013), and low nuclear Syk expression with chemo-resistance in patients treated with taxane-containing therapy (*P* = 0.006). Interestingly, despite the lack of association in the whole cohort, high nuclear Syk expression was significantly associated with better overall survival in certain subgroups (*P* = 0.001).

**Conclusions:**

The current study indicates a lack of correlation between calpain-2 expression and Syk and MAP4. Syk, MAP4 and calpain-1 appeared to significantly correlate with each other in the whole cohort, with calpain-1 being more highly associated with MAP4 and Syk in mucinous carcinomas. Overall, the current results suggest that Syk, MAP4, and calpain-1 expression are correlated with each other and these proteins may be involved in early stages of tumour spread.

**Electronic supplementary material:**

The online version of this article (10.1007/s00432-019-02856-9) contains supplementary material, which is available to authorized users.

## Introduction and aims

The calpain system has been confirmed to play an important role in influencing ovarian cancer patient outcomes with the adverse association between high calpain-2 expression and worse overall survival in ovarian cancer (Storr et al. 2012) being recently verified, and with calpain-4 and calpastatin expression also being found to be associated with overall survival (Zhang et al. 2018). However, the mechanisms whereby the calpain system exerts it important effects in ovarian cancer progression remain to be fully elucidated.

Spleen tyrosine kinase (Syk), a cytoplasmic non-receptor tyrosine kinase (Baldock et al. 2000), is widely expressed in a variety of hematopoietic cells (Singh et al. 2012; Krisenko and Geahlen 2015) and has been detected in osteoclasts, normal and tumourigenic mammary epithelial cells, melanocytes, human nasal fibroblasts, hepatocytes, neuronal cells and vascular endothelial cells suggesting that Syk has a general physiological function in a wide variety of non-hematopoietic cells (Coopman and Mueller 2006; Yanagi et al. 2001; Singh et al. 2012). Syk has been reported as a pro-survival factor in many hematopoietic malignancies (Krisenko and Geahlen 2015) and some epithelial original tumours (Geahlen 2014); however, it has also been hypothesised to have a tumour suppressor role in cancers of non-immune cells (Moroni et al. 2004; Coopman et al. 2000; Coopman and Mueller 2006; Krisenko and Geahlen 2015). A decrease in Syk expression in highly malignant and invasive tumour cells has been observed in a range of cancers including lung (Chuanliang et al. 2016) and hepatocellular cancer (Hong et al. 2012; Yuan et al. 2006). Expression has been found negatively associated with cell proliferation and anchorage-independent growth; moreover, negative associations have also been detected between Syk and cell migration, lymphovascular invasion, microvessel density and/or metastasis formation in cancers (Ogane et al. 2009; Fei et al. 2013; Nakashima et al. 2006; Coopman and Mueller 2006; Peng et al. 2013; Chuanliang et al. 2016).

Data in the literature have suggested potential interactions between Syk and the calpain system. A C-terminal fragment (40–45 kDa) of Syk was generated by calpain cleavage of human recombinant Syk (Baldock et al. 2000) and calpain inhibitors have been shown to significantly increase the presence of full-length Syk (72 kDa) on cell membrane (Gonscherowski et al. 2006) suggesting that Syk is a calpain substrate. An additional study suggests that Syk appears upstream of calpains rather than being a calpain substrate thus the effect of Syk on calpain activity seems contradictory and may depend on the cellular context (Fei et al. 2013).

A recent study suggested an association between Syk and MAP4 in ovarian cancer cells (Yu et al. 2015). MAP4 is the predominant human non-tubulin component of microtubule-associated protein in non-neuronal tissues (Orr et al. 2003). Syk-mediated tyrosine phosphorylation of MAP1B and MAP4 can induce paclitaxel resistance via reducing microtubule stabilisation (Yu et al. 2015; Krisenko et al. 2014; Wei and Birrer 2015).

As mentioned above, results from previous ovarian cancer studies confirm that the calpain system is associated with patient overall survival, particularly calpain-2 expression. In addition, calpain-1 expression showed significant association with tumour stage suggesting that calpain-1 might mediate tumour spread (Zhang et al. 2018). The current study sought to assess Syk and MAP4 expression in a large cohort of ovarian cancer specimens, to evaluate associations with patient clinical outcomes and other clinicopathological factors and additionally sought to determine whether there were any correlations between the expression of Syk, MAP4 and the calpain system.

## Materials and methods

### Clinical samples

The ovarian tissue microarray was composed of tumour cores from 575 ovarian cancer cases, with 448 chemo-naïve samples, as described previously (Zhang et al. 2018). The clinicopathological variables of the cohort are listed in Table 1. Patients were diagnosed with ovarian cancer and received treatment at Nottingham University Hospitals between 1991 and 2011. The majority of the patients (*n* = 357) received platinum-based chemotherapy among which 168 patients were treated with chemotherapy containing taxanes. Progression-free survival was defined as the length of time between start of treatment and clinical identification of recurrence. Data on resistance to chemotherapy was recorded, classified by the Gynaecologic Oncology Group (GOG) as either refractory (not responding to chemotherapy), resistant (an initial response to chemotherapy with recurrence within 6 months) or sensitive (when there was either no recurrence or recurred after 6 months). Ethical approval was obtained from Derbyshire Ethics Committee (07/H0401/156). This study is reported in accordance with REMARK (reporting recommendations for tumour marker prognostic studies) criteria (McShane et al. 2005).

**Table 1:** Clinicopathologic variables of the patient cohort

Variable	Number of patients	Percentage (%)
Age		
≤ 62	295	52.4
> 62	268	47.6
Histological subtypes		
HGSC	337	59.7
Mucinous carcinoma	60	10.6
Endometrioid carcinoma	68	12.1
CCC	53	9.4
LGSC	30	5.3
SBOT	15	2.7
Grade		
1	48	8.5
2	90	16.0
3	425	75.5
Stage		
I	203	36.7
II	64	11.6
III	245	44.3
IV	41	7.4
Residual disease		
No residual tumour	311	62.2
Residual tumour (< 2 cm)	58	11.6
Residual tumour (> 2 cm)	131	26.2
Adjuvant therapy		
Pt-based chemotherapy	357	63.3
Non-platinum-based chemotherapy	6	1.1
No chemotherapy	80	14.2
Information not available	121	21.5
Response to chemotherapy		
Refractory and resistance	66	17.7
Sensitivity	307	82.3
Progression status		
No recurrence	137	32.9
Recurred	280	67.1
Survival status		
Living	234	42.0
Deceased	323	58.0

### Tissue microarray, immunohistochemistry and interpretation

Protein expression was investigated using sections taken from the same tissue microarray blocks that were used in the previous study, that assessed calpain system protein expression, i.e., calpain-1, -2, -4 and calpastatin (Zhang et al. 2018). For the majority of the cases, a single tissue core was used for each patient. Fresh sections (4 µm) were cut from each block and placed on coated glass slides for the immunohistochemical assessment of protein expression.

Immunohistochemistry was performed as described previously (Storr et al. 2012; Zhang et al. 2018). Primary antibodies were diluted in Bond Primary Antibody Diluent (Leica, Denmark), MAP4 antibody (1:400; Bethyl Laboratories, INC; A301-489A) and Mouse monoclonal anti-human Syk antibody (4D10.1) (1:1600; Thermo Fisher Scientific; MA5-13087), and applied to the tissue overnight at 4 °C. The specificity of these antibodies was initially confirmed by Western blotting before conducting IHC (Fig. S1). Negative controls omitted primary antibody. Slides were scanned and cytoplasmic staining intensity semi-quantitatively assessed, for Syk and MAP4, using an immunohistochemical H-score as described previously (Storr et al. 2012; Zhang et al. 2018). Nuclear staining intensity was also scored for Syk, and the percentage of stained nuclei recorded. Greater than 25% of the slides were examined by a second independent assessor blinded to scores and clinicopathologic criteria. Single measure intraclass correlation coefficient (ICC) analysis was used to determine the level of agreement between independent scorers. The single measure ICCs between scores were 0.925, 0.833 and 0.887 for anti-Syk antibody cytoplasmic and nuclear-stained samples, and anti-MAP4 antibody-stained samples, respectively. A non-biased cut-point of the immunohistochemical scores, to dichotomise data, was determined using X-tile software using patient OS (Camp et al. 2004; Storr et al. 2012).

### Statistical analysis

The relationships between categorised protein expression and clinicopathologic factors were examined using Pearson’s Chi-squared test of association (*χ*^2^) or Fisher’s exact test if a cell count was less than 5 in a 2 × 2 table. To assess the relationship between protein expression and survival outcomes, survival curves were generated using the Kaplan–Meier method and statistical significance determined by the Log-rank test. Multivariate survival analysis was performed using a proportional hazards model by Cox regression analysis to estimate hazard ratios and 95% confidence intervals for overall survival. Spearman’s rank correlation coefficient (Spearman’s *ρ*) test was performed to assess the correlation between the expression levels of different proteins. The correlation strength was interpreted as follows: Spearman *ρ* (rs) less than 0.16 is too weak to be meaningful, ranging from 0.16 to 0.19 as very weak correlation; 0.20 to 0.39 as a weak correlation; 0.40 to 0.59 as a moderate correlation; 0.60 to 0.79 as strong correlation and 0.80 or greater as very strong correlation (Divaris et al. 2012). Statistical analyses were carried out using SPSS 22.0 software. *P* values < 0.05 were considered statistically significant.

## Results

**Fig. 1:**
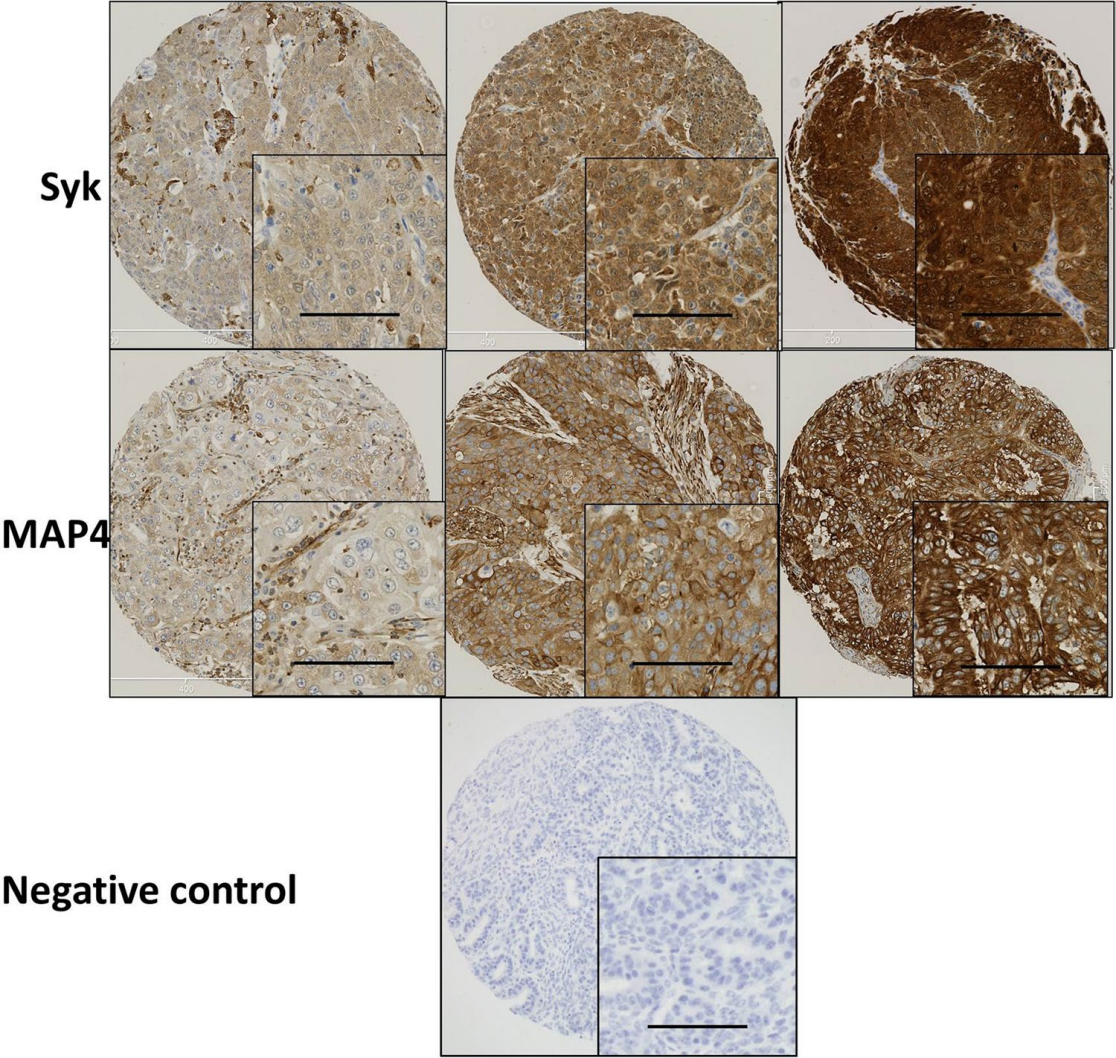
Representative photomicrographs of Syk and MAP4 expression in ovarian carcinoma cells. Expression levels, including low (left), medium (middle) and high (right) staining, of Syk and MAP4 at × 10 magnification with × 20 magnification inset panel. Negative controls omitted primary antibody. Scale bar represents 100 µm

Similar to reports by Zhang et al. (2009) and Fei et al. (2013) Syk was expressed in both the cytosol and nucleus. MAP4 showed predominant staining in the cytoplasm of the ovarian cancer cells with membranous staining apparent. Representative staining patterns of weak, moderate, and strong staining intensity are shown in Fig. 1. Both Syk and MAP4 showed granular/diffuse staining with heterogeneity between adjacent cancer cells. In addition to the staining of tumour cells, Syk and MAP4 staining was observed in areas of tumour-adjacent normal tissue; moreover, Syk staining was also observed in immune cells. The current study focused only on the staining of tumour cells.

### The expression of Syk and MAP4 and clinicopathological factors

High cytoplasmic Syk (Syk-c) expression was observed in 374 (85%) out of 441 cases, and nuclear Syk (Syk-n) expression was observed in 329 (75%) cases (Syk-c cut-point: 65; Syk-n cut-point: 12). Pearson’s Chi-squared test was performed to evaluate the relationships with the expression of calpain-system proteins and with clinicopathologic characteristics, with results shown in Table 2. HGSCs often displayed higher Syk expression in the cytoplasm and nucleus (Syk-c: *χ*^2^ = 58.835, *df* = 5, *P* = 2.115E−11 and Syk-n: *χ*^2^ = 29.874, *df* = 5, *P* = 0.000016, respectively). Low Syk-c expression was linked with lower stage (*χ*^2^ = 10.725, *df* = 3, *P* = 0.013), whilst low Syk-n expression significantly associated with chemo-resistance in patient treated by therapy containing taxanes (*χ*^2^ = 7.582, *df* = 1, *P* = 0.006).

**Table 2:** Association between Syk expression and clinicopathological criteria

Variable	Syk-c expression				Syk-n expression				
	Low	High		*P* value	Low		High		*P* value
Age									
≤ 62	30 (6.8%)	184 (41.8%)	0.492		51 (11.6%)			163 (37.0%)	0.447
> 62	37 (8.4%)	189 (43.0%)			61 (13.9%)			65 (37.5%)	
Histological subtypes									
HGSC	25 (5.7%)	247 (56.0%)	**2.115E**−11*		57 (12.9%)		215 (48.8%)		**1.6E**−5*
Mucinous	9 (2.0%)	34 (7.7%)			10 (2.3%)		33 (7.5%)		
Endometrioid	9 (2.0%)	45 (10.2%)			16 (3.6%)		38 (8.6%)		
CCC	23 (5.2%)	21 (4.8%)			25 (5.7%)		19 (4.3%)		
LGSC	1 (0.2%)	17 (3.9%)			4 (0.9%)		14 (3.2%)		
SBOT	0 (0.0%)	10 (2.3%)			0 (0.0%)		10(2.3%)		
Grade									
1	4 (0.9%)	29 (6.6%)	0.813		7 (1.6%)		26 (5.9%)		0.174
2	9 (2.0%)	56 (12.7%)			11 (2.5%)		54 (12.2%)		
3	54 (12.2%)	289 (65.5%)			94 (21.3%)		249 (56.5%)		
Stage									
I	36 (8.3%)	123 (28.3%)	0.013*		50 (11.5%)		109 (25.1%)		0.209
II	6 (1.4%)	43 (9.9%)			12 (2.8%)		37 (8.5%)		
III	20 (4.6%)	175 (40.3%)			42 (9.7%)		153 (35.3%)		
IV	5 (1.2%)	26 (6.0%)			8 (1.8%)		23 (5.3%)		
Residual disease									
No residual tumour	44 (11.3%)	195 (50.0%)	0.227		72 (18.5%)		167 (42.8%)		0.076
Residual tumour (< 2 cm)	5 (1.3%)	41 (10.5%)			10 (2.6%)		36 (9.2%)		
Residual tumour (> 2 cm)	13 (3.3%)	92 (23.6%)			20 (5.1%)		85 (21.8%)		
Response to platinum-based chemotherapy									
Chemo-refractory	6 (2.1%)	24 (8.5%)	0.589*		13 (4.6%)	17 (6.0%)			0.154
Chemo-resistance	2 (0.7%)	19 (6.7%)			6 (2.1%)	15 (5.3%)			
Chemo-sensitivity	35 (12.4%)	196 (69.5%)			61 (21.6%)	170 (60.3%)			
Response to taxane-containing (i.e., paclitaxel, docetaxel) chemotherapy regimens									
Chemo-resistance	3 (2.5%)	18 (14.8%)	0.774*		11 (9.0%)	10 (8.2%)			**0.006**
Chemo-sensitivity	17 (13.9%)	84 (68.9%)			23 (18.9%)	78 (63.9%)			

*HGSC* high-grade serous carcinoma, *LGSC* low-grade serous carcinoma, *CCC* clear-cell carcinoma, *SBOT* serous borderline ovarian tumour

*Expected count less than 5. Significant *P* values are indicated by bold font

The above analysis was also conducted in chemotherapy-naïve cases (*n* = 448) (data not shown). Significant associations were found between Syk expression and ovarian cancer subtype (Syk-c: *χ*^2^ = 46.443, *df* = 5, *P* = 7.3773E−9; Syk-n: *χ*^2^ = 26.744, *df* = 5, *P* = 0.000064), stage (only Syk-c: *χ*^2^ = 11.520, *df* = 3, *P* = 0.009), and tumour response to taxane-containing chemotherapy (only Syk-n: *χ*^2^ = 7.078, *df* = 1, *P* = 0.008).

High MAP4 expression was observed in 291 (66%) out of 442 cases (cut-point: 152). Pearson’s Chi-squared test was performed to evaluate the associations of clinicopathological factors with MAP4 expression (Table 3). HGSCs exhibited significantly higher MAP4 expression (*χ*^2^ = 56.386, *df* = 5, *P* = 6.7681E−11). Low expression was significantly associated with lower stage (*χ*^2^ = 15.857, *df* = 3, *P* = 0.001) and lower grade (*χ*^2^ = 22.933, *df* = 2, *P* = 0.00001). A significant association was detected between high MAP4 expression and the presence of residual tumour (*χ*^2^ = 10.426, *df* = 2, *P* = 0.005).

**Table 3:** Association between MAP4 expression and clinicopathological criteria

Variable	MAP4		
	Low	High	*P* value
Age			
≤ 62	82 (18.6%)	136 (30.8%)	0.169
> 62	70 (15.9%)	153 (34.7%)	
Histological subtypes			
HGSC	59 (13.3%)	211 (47.7%)	**6.7681E**−**11**
Mucinous	25 (5.7%)	16 (3.6%)	
Endometrioid	22 (5.0%)	33 (7.5%)	
CCC	29 (6.6%)	16 (3.6%)	
LGSC	11 (2.5%)	8 (1.8%)	
SBOT	6 (1.4%)	6 (1.4%)	
Grade			
1	16 (3.6%)	19 (4.3%)	**0.00001**
2	38 (8.6%)	28 (6.3%)	
3	97 (22.0%)	243 (55.1%)	
Stage			
I	75 (17.2%)	89 (20.5%)	**0.001**
II	12 (2.8%)	36 (8.3%)	
III	52 (12.0%)	140 (32.2%)	
IV	10 (2.3%)	21 (4.8%)	
Residual disease			
No residual tumour	97 (24.8%)	144 (36.8%)	**0.005**
Residual tumour (< 2 cm)	10 (2.6%)	38 (9.7%)	
Residual tumour (> 2 cm)	27 (6.9%)	75 (19.2%)	
Response to platinum-based chemotherapy			
Chemo-refractory	8 (2.8%)	22 (7.8%)	0.509
Chemo-resistance	8 (2.8%)	11 (3.9%)	
Chemo-sensitivity	72 (25.5%)	161 (57.1%)	
Response to taxane-containing (i.e., paclitaxel, docetaxel) chemotherapy regimens			
Chemo-resistance	9 (7.5%)	10 (8.3%)	0.072
Chemo-sensitive	27 (22.5%)	74 (61.7%)	

*HGSC* high-grade serous carcinoma, *LGSC* low-grade serous carcinoma, *CCC* clear-cell carcinoma, *SBOT* serous borderline ovarian tumour

*Expected count less than 5. Significant *P* values are indicated by bold type

The above analysis was also conducted in chemotherapy-naïve cases (*n* = 448). MAP4 expression was significantly associated with subtype (*χ*^2^ = 45.528, *df* = 5, *P* = 1.1332E−8), grade (*χ*^2^ = 14.209, *df* = 2, *P* = 0.001), stage (*χ*^2^ = 15.050, *df* = 3, *P* = 0.002), residual disease (*χ*^2^ = 9.965, *df* = 1, *P* = 0.007) and tumour response to taxane-containing chemotherapy (*χ*^2^ = 4.596, *df* = 1, *P* = 0.032; high MAP4 expression associated with chemo-sensitisation).

Patients were then grouped according to whether they had an organ-confined tumour (i.e., group 1: stage 1 and group 2: stage 2–4) or according to whether they had distant metastasis (i.e., group 1: stage 1–3 and group 2: stage 4). Higher significance was observed for Syk and MAP4 expression, regarding tumour spread, when grouping patients into two broader categories by organ-confined status than grouping by FIGO staging classifications (Table S1). Low Syk-c, low Syk-n and low MAP4 showed significant association with organ-confined status of tumour (Syk-c: *χ*^2^ = 9.975, *df* = 1, *P* = 0.002; Syk-n: *χ*^2^ = 4.169, *df* = 1, *P* = 0.041; and MAP4: *χ*^2^ = 15.402, *df* = 1, *P* = 0.000087), whereas no significant association was detected between protein expression and cancer distant metastasis. In chemotherapy-naïve cases (*n* = 448), low Syk-c, low Syk-n and low MAP4 also showed significant association with organ-confined status of tumour (Syk-c: *χ*^2^ = 10.551, *df* = 1, *P* = 0.001; Syk-n: *χ*^2^ = 5.041, *df* = 1, *P* = 0.025; MAP4: *χ*^2^ = 14.322, *df* = 1, *P* = 0.000154). Such findings suggest that, similar to previous observations with calpain-1, MAP4 and Syk expression may be more related to early events of tumour spread.

### The expression of Syk and MAP4 and clinical outcomes

**Fig. 2:**
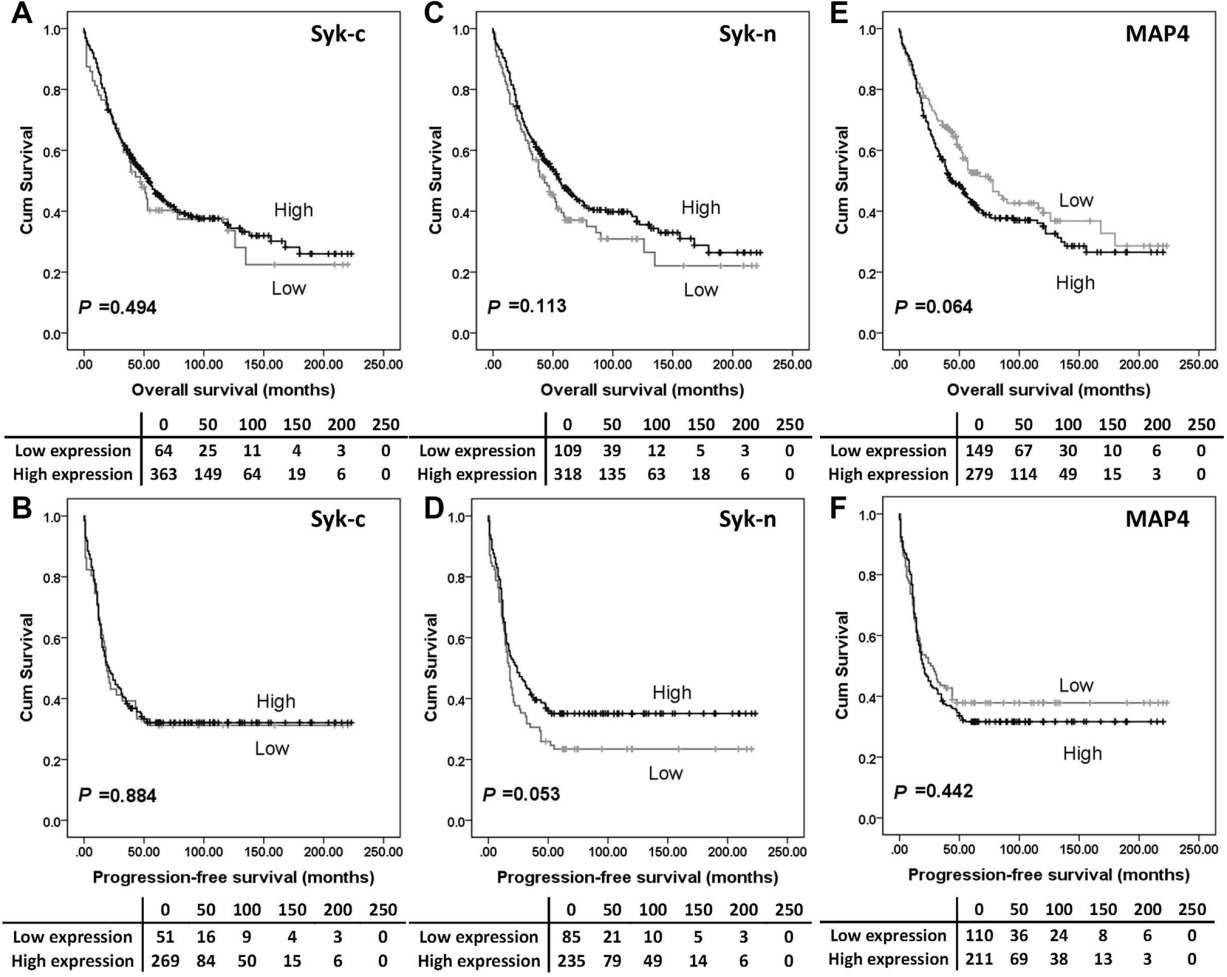
Kaplan–Meier survival curves show the impact of Syk-c, Syk-n and MAP4 expression on overall survival and progression-free survival. There were no differences in survival between patients with tumours expressing high MAP4 or Syk and those with tumours expressing low MAP4 or Syk. Significance was determined using the log-rank test. The tables shown below the Kaplan–Meier survival curves listed the number of patients at risk at the specific months. High expression—black line, low expression—grey line

**Fig. 3:**
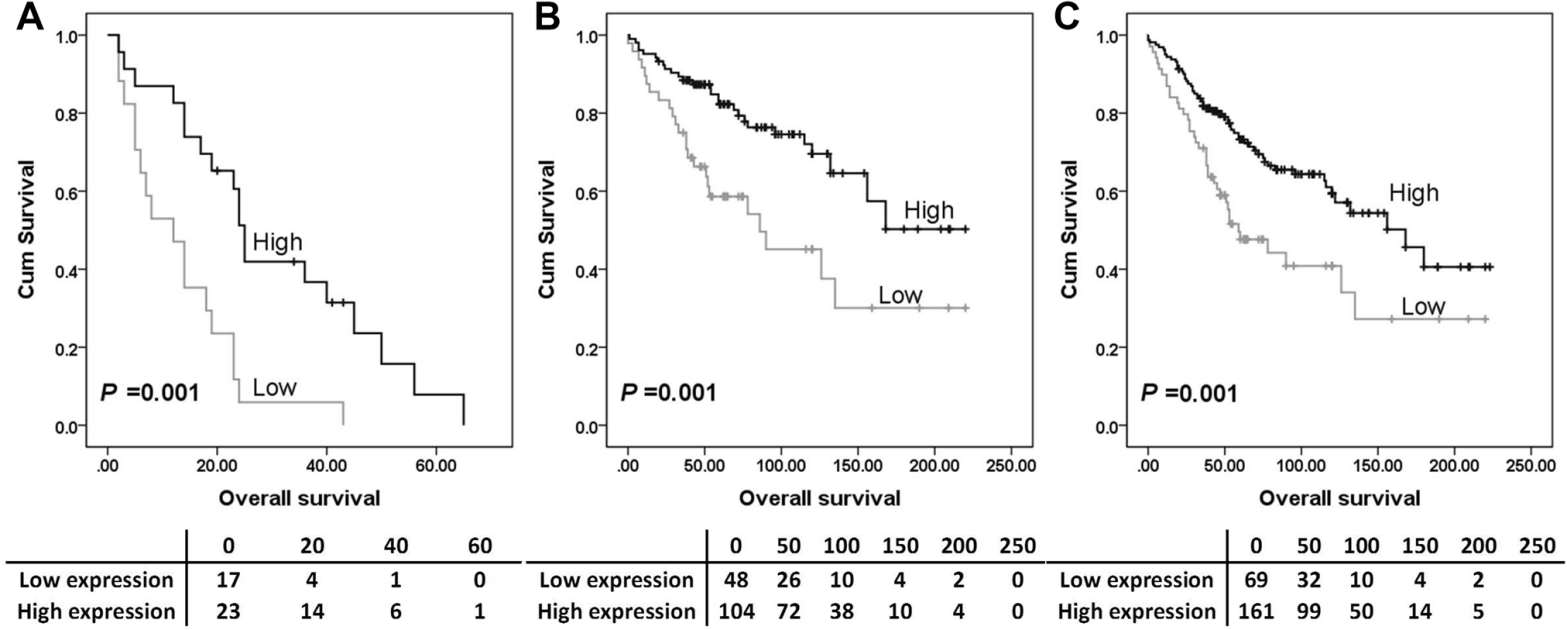
Kaplan–Meier survival curves show the impact of Syk expression on overall survival in different subgroups. Survival analysis shows that high Syk-n expression significantly associated with better (*P* = 0.010) overall survival for ovarian cancer patients resistant to platinum-based chemotherapy (**a**), patients with tumour confined to the ovaries (**b**) and patients with no residual carcinoma (**c**). Significance was determined using the log-rank test. The tables shown below the Kaplan–Meier survival curves listed the number of patients at risk at the specific months. High expression—black line, low expression—grey line

The expression of Syk-c, -n or MAP4 had no significant associations with overall survival (Fig. 2a, c and e) or progression-free survival (Fig. 2b, d and f). Further analysis was conducted to investigate the prognostic significance of Syk and MAP4 in the individual subgroups defined by clinicopathologic variables. *P* values of log-rank tests for overall survival in association with each protein expression are listed in Table S2. High Syk-n expression indicated a better overall survival among platinum-resistant patients (*P* = 0.001, Fig. 3a), patients with tumours confined to the ovaries (stage 1 ovarian cancer) (*P* = 0.001, Fig. 3b) and patients with no residual disease (*P* = 0.001) (Fig. 3c).

### Correlations between calpain family expression and calpain-associated protein expression

Correlations between Syk and MAP-4 expression with calpain system proteins (i.e., calpain-1, -2, -4 and calpastatin) were studied in the whole cohort. The correlation strength between calpain-2 expression and Syk and MAP4 expression are considered too weak to be meaningful (rs < 0.16). Nevertheless, MAP4 expression was moderately correlated with calpain-1 (rs = 0.537, *P* < 0.001) (Table 4); in addition, Syk-c and Syk-n expression were strongly associated with each other (rs = 0.809, *P* < 0.001) (Table 4).

**Table 4:** Spearman’s rank correlation coefficient between defined proteins of interest (469 valid cases)

	Calpastatin	Calpain-1	Calpain-2	Calpain-4
Syk-c				
rs	0.135*	0.396*	0.127*	0.145*
Sig.	0.006	4.3041E−17	0.010	0.003
*N*	415	416	415	416
Syk-n				
rs	0.160*	0.346*	0.082	0.129*
Sig.	0.001	4.0912E−13	0.097	0.009
*N*	415	416	415	416
MAP4				
rs	0.244*	0.537*	0143*	0.286*
Sig.	4.8817E−7	2.3144E−32	0.004	2.8108E−9
*N*	414	415	414	415

	Syk-c	Syk-n
MAP4		
rs	0.385*	0.290*
Sig.	1.350E−16 (*n* = 429)	9.765E−10 (*n* = 429)
Syk-c		
rs	–	0.809*
Sig.	–	3.189E−103 (*n* = 441)

*Correlation is significant at the 0.01 level (two-tailed)

Significant *P* values are indicated by bold font

*rs* Spearman’s correlation coefficient, *Syk-n* nuclear Syk, *Syk-c* cytoplasmic Syk

The correlations were also studied in each histological subtype separately (data not shown). The moderate correlation between calpain-1 and MAP4 was also observed in HGSCs (rs = 0.472, *P* < 0.001, *n* = 255) and CCCs (rs = 0.516, *P* < 0.001, *n* = 42), whilst strong correlation was observed in endometrioid (rs = 0.687, *P* < 0.001, *n* = 50) and mucinous tumours (rs = 0.708, *P* < 0.001, *n* = 36), with no significant correlation detected between calpain-1 and MAP4 in LGSC. For calpain-1 and Syk expression, a weak correlation was found in the whole cohort and also in HGSCs (Syk-c: rs = 0.347, *P* < 0.001, *n* = 257; Syk-n: rs = 0.313, *P* < 0.001, *n* = 257) and CCCs (Syk-c: rs = 0.378, *P* = 0.014, *n* = 42; Syk-n: rs = 0.327, *P* = 0.035, *n* = 42), whilst a moderate correlation was found in mucinous tumours (Syk-c: rs = 0.425, *P* = 0.007, *n* = 39; Syk-n: rs = 0.471, *P* = 0.002, *n* = 39), no correlation was detected between calpain-1 and Syk expression in LGSCs or endometrioid tumours. Either no correlation or weak correlation was detected between Syk-n and MAP4 expression in the whole cohort and in the five groups of histological subtypes (data not shown). Between Syk-c and MAP4 expression, a weak correlation was found in the whole cohort and also in HGSCs (rs = 0.312, *P* < 0.001, *n* = 265) and mucinous tumours (rs = 0.376, *P* = 0.018, *n* = 39), whilst a moderate correlation was found in CCCs (rs = 0.576, *P* < 0.001, *n* = 44); no correlation was detected between Syk and MAP4 expression in LGSCs or endometrioid tumours (data not shown). It is noticeable that CXCR3 and Syk expression lacked correlation or was weakly correlated in the whole cohort and 4 of the histological subtypes; only in LGSC type, strong correlation was detected (Syk-c: rs = 0.640, *P* = 0.006, *n* = 17; Syk-n: rs = 0.606, *P* = 0.010, *n* = 17). Findings suggest that the role this panel of proteins played and how they cooperated with each other may be histological subtype dependent.

Additional survival analysis was conducted by recategorised tumours according to expression of any two of MAP4, Syk-c, Syk-n, calpain-1, -2, -4 and calpastatin into four groups each time; for example, grouping patients into the following four groups (i.e., high Syk-n and high Syk-c; high Syk-n and low Syk-c; low Syk-n and high Syk-c; low Syk-n and low Syk-c). On the survival analysis, the *P* values from the log-rank test obtained with recategorised groups were less significant than those obtained with single marker (Table S3).

## Discussion

Among the ovarian cancer histological subtypes, HGSCs displayed higher expression of Syk-c and -n (Syk-c: *χ*^2^ = 58.835, *df* = 5, *P* = 2.115E−11 and Syk-n: *χ*^2^ = 29.874, *df* = 5, *P* = 0.000016, respectively). In addition, the present study indicated that low Syk-c expression was associated with lower tumour stage (*χ*^2^ = 10.725, *df* = 3, *P* = 0.013). In light of such data, it is likely that Syk may promote tumour spread via increasing cell invasion and migration at the early stage of ovarian carcinogenesis. Consistently, high Syk expression (mRNA level, *n* = 45; protein level, *n* = 38) significantly correlated with worse survival, enhanced cell migration and metastases to the lymph nodes in patients with squamous cell carcinomas of the head and neck (Luangdilok et al. 2007). Other pro-survival functions of Syk include anti-apoptosis (Geahlen 2014; Krisenko and Geahlen 2015), cancer cell growth and survival (Prinos et al. 2011; Udyavar et al. 2013; Fei et al. 2013), migration and dissemination (Luangdilok et al. 2007; Ghotra et al. 2015; Katz et al. 2005; Krisenko and Geahlen 2015). Yet, other studies suggest that Syk is absent from many highly aggressive epithelial cell-derived tumours (Krisenko and Geahlen 2015; Coopman and Mueller 2006) and Syk may increase cell–cell interactions and limit EMT (Krisenko and Geahlen 2015). To be specific, Syk knockdown in more well-differentiated cancers was found to enhance invasive/anchorage-independent growth and motility (Fei et al. 2013) whilst its re-introduction in more malignant, invasive cancer cells decreased cancer malignancy through increasing adhesion and reducing tumour cell growth, motility, invasion and metastasis (Ogane et al. 2009; Fei et al. 2013; Peng et al. 2013; Krisenko and Geahlen 2015). The invasion suppressive function of full-length Syk has been reported to correlate with Syk nuclear localisation suggesting that Syk-n possesses biological activities associated with tumour suppression in mammary epithelial cells (Wang et al. 2003).

In the current study, a tumour-suppressive effect of Syk-n was reflected in its negative association with chemo-resistance in patients treated by therapy containing taxane (*χ*^2^ = 7.582, *df* = 1, *P* = 0.006) and positive association with overall survival when patients had tumours confined to the ovaries (stage 1 ovarian cancer) (*P* = 0.001), in the subgroup of platinum-resistant patients (*P* = 0.001) and in the group of patients with no residual disease (*P* = 0.001). Current findings are in contrast to those from a previous study conducted in ovarian cancer cells where Syk expression/activity levels positively associated with cell resistance to paclitaxel; moreover, in the same study, pharmacological and genomic Syk inhibitors had a synergistic effect with paclitaxel both in vitro and in vivo especially in cells that were resistant to paclitaxel (Yu et al. 2015). A separate study showed that Syk was preferentially expressed in recurrent HGSCs after chemo-therapeutic treatment in comparison with the primary tumours from the same patients (Jinawath et al. 2010). Moreover, restriction of Syk to the nucleus has been suggested to diminish stress-induced activation of caspase 3 in B cells (Zhou et al. 2006; Mohammad et al. 2016) and tumour cells (Wang et al. 2005).

The discrepancy between the above and the strong and positive correlation between Syk-c and -n expression in the current study (rs = 0.809, *P* < 0.001) may be due to the different splice variants of Syk and their localisation. Although Syk(L) and Syk(S) cannot be discriminated via IHC with the antibody used in the current study, which targets amino acids 313–339, Syk-n and Syk-c were studied separately. Full-length Syk is known as Syk long isoform, Syk(L), whilst the shorter gene product Syk(S) omits a stretch of 23 amino acids (AA283–305) in linker B (Grädler et al. 2013; Krisenko and Geahlen 2015). Syk(L) and Syk(S) were found in both nucleus and cytoplasm (Wang et al. 2003; Zhou et al. 2006; Luangdilok et al. 2007) with Syk(L) predominantly presenting in the nucleus and Syk(S) in cytoplasm in untreated tumour cells (Prinos et al. 2011; Wang et al. 2005). A Syk splicing pattern was linked to ovarian but not breast cancer (Klinck et al. 2008; Venables et al. 2008; Prinos et al. 2011) and SKOV3 cells reportedly preferentially express Syk(L) (Prinos et al. 2011). Further investigation is needed to understand the contradictory functions of Syk in terms of cellular context and tumour microenvironment.

In the current study, a significant association was detected between high MAP4 expression and the presence of residual tumour (*χ*^2^ = 10.426, *df* = 2, *P* = 0.005). Low MAP4 expression was significantly associated with lower stage (*χ*^2^ = 15.857, *df* = 3, *P* = 0.001) which agreed with a study of esophageal squamous cell carcinomas (*n* = 364), where MAP4 expression was linked with tumour stage and lymph node metastasis (Jiang et al. 2016). In addition, the knockdown of MAP4 was found able to markedly inhibit bladder cancer cells invasion (Ou et al. 2014). In addition, in the present study, low MAP4 expression was significantly associated with lower grade (*χ*^2^ = 22.933, *df* = 2, *P* < 0.001) which has also been observed in bladder cancer (*n* = 34) (Ou et al. 2014). Although a higher level of MAP4 mRNA was found in non-small cell lung carcinomas when compared to normal lung tissues (Cucchiarelli et al. 2008) and MAP4 expression was associated with shorter survival of the esophageal squamous cell carcinoma patients (*n* = 364) (Jiang et al. 2016), no significant association was detected in the present study between MAP4 expression and overall survival in either the whole cohort or any of the subgroups tested.

An association between MAP4 and paclitaxel resistance has been observed in different cancer types including ovarian cancer (Poruchynsky et al. 2001; Orr et al. 2003; Aoki et al. 2009). In the chemotherapy-naïve cases of the current study, the correlation between high MAP4 expression and chemo-sensitisation was statistically significant in patients treated by taxane-containing chemotherapy (*χ*^2^ = 4.596, *df* = 1, *P* = 0.032). As an anti-tumour drug, taxane induces microtubule stabilisation and reduces microtubule dynamics which promotes mitotic arrest and cell death (Aoki et al. 2009). Based on the mechanism of taxane on tumour cells, proteins that are involved in the regulation of cellular microtubule dynamics via interacting with tubulin dimers or polymerised microtubules clearly possess the potential for modulating the cell sensitivity towards paclitaxel (Orr et al. 2003). The drug sensitivity profile of ovarian cancer paclitaxel-resistant sublines parallels the regulation of MAP4 modification: MAP4 phosphorylation and dissociation from microtubules decrease the sensitivity of paclitaxel-resistant ovarian cell lines towards paclitaxel (Poruchynsky et al. 2001; Orr et al. 2003). However, down-regulation of MAP4 in SKOV3 cells did not influence the sensitivity to either paclitaxel or cisplatin; and MAP4 status was not correlated with the survival of patients treated with either taxane-based regimen or taxane-free regimen (Aoki et al. 2009). The precise role for MAP4 in tumour sensitivity to taxanes remains to be defined.

As indicated above, results from previous ovarian cancer studies confirm that calpain-2 is an important factor in cancer survival (Storr et al. 2012; Zhang et al. 2018). In addition, calpain-4 and calpastatin were also associated with patient overall survival (Zhang et al. 2018). Such previous studies focused on protein expression and thus cannot directly reflect the actual enzyme activity level of the calpain system. How the calpain system is involved in patient outcomes remains unclear. With low calpain-1 expression associating with stage 1 tumour (Zhang et al. 2018), in the current study, low Syk-c and MAP4 expression also related to organ-confined status (Syk-c: *χ*^2^ = 9.975, *df* = 1, *P* = 0.002; and MAP4: *χ*^2^ = 15.402, *df* = 1, *P* = 0.000087) (Tables 2, 3). Thus, the calpain system, together with Syk-c and MAP4, might be linked with overall survival via their associations with tumour spread. Currently, there is a lack of supporting information to explain the positive correlations between calpain-1 and Syk/MAP4 (MAP4 and calpain-1: rs = 0.537, *P* = 2.3144E−32; Syk-c and calpain-1: rs = 0.396, *P* = 4.3041E−17; MAP4 and Syk-c: rs = 0.385, *P* = 1.350E−16) (Table 4). In vitro studies focusing on the activity of these proteins and ovarian cancer cell invasion might shed more light on whether, and how, the calpain system interacts with Syk/MAP4 to influence patient outcome.

In conclusion, both MAP4 and Syk were associated with tumour stage. The current study also observed the paradoxical roles of Syk. High Syk-n expression indicated a better overall survival in certain subgroups of patients. Low Syk-n expression significantly associated with chemo-resistance in patients treated by therapy containing taxane which support the role of Syk as a tumour suppressor. Contradictory, low Syk-c expression was associated with low stage. Together, it appears from this study of ovarian cancers that high Syk expression may facilitate tumour spreading (higher stage) whilst high Syk-n sensitises tumour cells to taxane-containing chemotherapy. Significant correlations between MAP4, Syk, and calpain-1 suggest that calpain, Syk and MAP4 might be interrelated and interact on each other to modulate cancer cell spread. How MAP4, Syk and calpain-1 are involved in tumour cell spreading/migration, and in cellular response to taxane-containing chemotherapy, remains to be elucidated.

## Electronic supplementary material

Below is the link to the electronic supplementary material.

Supplementary material 1 (PDF 388 KB)

## Abbreviations

MAP: Microtubule-associated protein
Syk: Spleen tyrosine kinase
Syk-c: Cytoplasmic Syk
Syk-n: Nuclear Syk
