# Supplementary material for: Expression of Syk and MAP4 proteins in ovarian cancer
Source: J Cancer Res Clin Oncol. 2019 Feb 8;145(4):909–19. doi: 10.1007/s00432-019-02856-9 (PMC6435630; doi:10.1007/s00432-019-02856-9)
Supplement: Supplementary file 1 — Supplementary material 1 (PDF 388 KB) [file 432_2019_2856_MOESM1_ESM.pdf]

Expression of Syk and MAP4 proteins in ovarian cancer

Siwei Zhang[1], Suha Deen[2], Sarah J Storr[1], Anqi Yao[1], Stewart G Martin[1,3]

1 Academic Clinical Oncology, Nottingham Breast Cancer Research Centre, School of Medicine, University of Nottingham, Nottingham University Hospitals NHS Trust, City Hospital Campus, Nottingham, NG5 1PB, UK.

2 Department of Pathology, Queen's Medical Centre, Nottingham University Hospital, Nottingham, NG7 2UH, UK

3 Corresponding author: Academic Clinical Oncology, Nottingham Breast Cancer Research Centre, School of Medicine, University of Nottingham, Nottingham University Hospitals NHS Trust, City Hospital Campus, Nottingham, NG5 1PB, UK

Telephone: +44(0)115 823 1846

Email: stewart.martin@nottingham.ac.uk.

Supplements

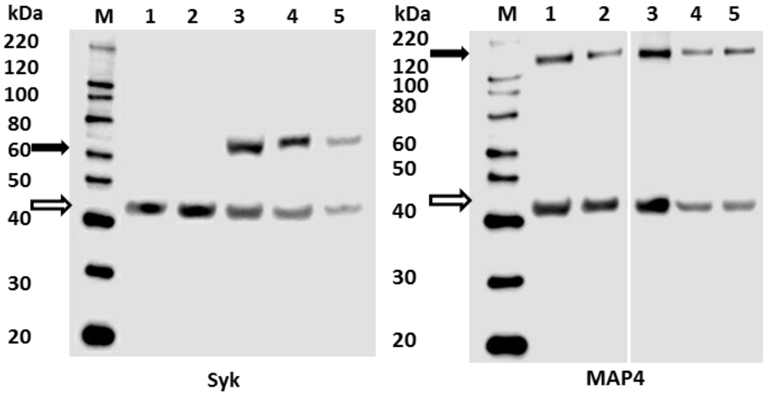

Fig. S1 The specificity of anti-Syk and anti-MAP4 antibody. Syk and MAP4 expression across the panel of ovarian cancer cell lines were detected by Western blotting analysis. Primary antibodies were diluted in 1:1000 dilution. A single band corresponding to the molecular mass of the intended target was observed in the Western blotting studies which indicated the specificity of both anti-MAP4 (BETHYL LABORATORIES, UK) and anti-Syk (Thermo Fisher SCIENTIFIC, UK) antibody. Figures present the representative blots of 2 independent experiments with lysates from cells with different passage numbers. Open arrows indicate β-actin (42 kDa) which was used as the loading control. Black arrows indicate Syk (72 kDa) and MAP4 (210 kDa), respectively. Lane M: Protein marker, Lane 1: A2780, Lane 2: A2780-cis, Lane 3: SKOV3, Lane 4: PEO1, and Lane 5: PEO4.

Table S1 The association between protein expression and tumour stage

Variable	Syk-c			Syk-n			MAP4		
	Low	High	P-value	Low	High	P-value	Low	High	P-value
**Stage**									
**Tumour confined to ovaries (stage 1)**	36(8.3%)	123 (28.3%)	**0.002**	50 (11.5%)	109 (25.1%)	**0.041**	75 (17.2%)	89 (20.5%)	**0.000087**
**Tumour spread out of ovaries (stage 2, 3 and 4)**	31 (7.1%)	244 (56.2%)		62 (14.3%)	213 (49.1%)		74 (17.0%)	197 (45.3%)	
**Stage**									
**Without distant metastasis (stage 1,2 and 3)**	62 (14.3%)	341 (78.6%)	0.912	104 (24.0%)	299 (68.9%)	1.000	139 (32.0%)	265 (60.9%)	0.808
**Distant metastasis occurred (stage 4)**	5 (1.2%)	26 (6.0%)		8 (1.8%)	23 (5.3%)		10 (2.3%)	21 (4.8%)	

Significant *P* values are indicated by bold font.

Table S2 P values from the log-rank test of overall survival in different subgroups

Variables	*P* value		
	MAP4	Cytoplasmic Syk	Nuclear Syk
**Histological subtypes**			
**High grade serous carcinoma**	0.699.	0.581	0.506
**Mucinous carcinoma**	0.791	0.026	0.115
**Endometrioid carcinoma**	0.110	0.188	0.006
**Clear-cell carcinoma**	0.988	0.822	0.453
**Low grade serous carcinoma**	0.212	0.549	0.196
**Stage**			
**Stage 1 (confined tumour) (n=152)**	0.679	0.002	**0.001**
**Stage 2, 3 & 4 (tumour spread) (n=269)**	0.404	0.688	0.224
**Stage**			
**Stage 1, 2 & 3 (without distant metastasis) (n=390)**	0.104	0.568	0.107
**Stage 4 (with distant metastasis) (n=31)**	0.061	0.402	0.509
**Sensitivity to platinum-based chemotherapy**			
**Sensitive (n=195)**	0.885	0.486	0.464
**Resistant (n=40)**	0.996	0.016	**0.001**
**Residual disease**			
**No residual tumour (n=232)**	0.867	0.054	**0.001**
**residual tumour (n=147)**	0.540	0.349	0.571

Significant *P* values are indicated by bold font.

Table S3 P values from the log-rank test of overall survival related to combinational protein expression.

	**Syk-c (*P*=0.494)**	**Syk-n (*P*=0.113)**	**MAP4 (*P*=0.064)**	**CXCL4 (*P*=0.031)**	**CXCR3 (*P*=0.182)**
**Calpastatin (*P*=0.010)**	0.174	0.178	0.024	0.225	0.188
**Calpain-1 (*P*=0.153)**	0.273	0.292	0.283	0.309	0.445
**Calpain-2 (*P*=0.026)**	0.087	0.056	0.037	0.285	0.152
**Calpain-4 (*P*=0.003)**	0.139	0.061	0.034	0.074	0.068
**Syk-c (*P*=0.494)**	\	0.424	0.220	0.358	0.468
**Syk-n (*P*=0.113)**	\	\	0.112	0.166	0.208
**MAP4 (*P*=0.064)**	\	\	\	0.084	0.097
